# Optimizing the Detection of Wakeful and Sleep-Like States for Future Electrocorticographic Brain Computer Interface Applications

**DOI:** 10.1371/journal.pone.0142947

**Published:** 2015-11-12

**Authors:** Mrinal Pahwa, Matthew Kusner, Carl D. Hacker, David T. Bundy, Kilian Q. Weinberger, Eric C. Leuthardt

**Affiliations:** 1 Department of Biomedical Engineering, Washington University, St. Louis, Missouri, United States of America; 2 Department of Computer Science and Engineering, Washington University, St. Louis, Missouri, United States of America; 3 School of Medicine, Washington University, St. Louis, Missouri, United States of America; 4 Department of Neurological Surgery, Washington University, St. Louis, Missouri, United States of America; 5 Center for Innovation in Neuroscience and Technology, Washington University, St. Louis, Missouri, United States of America; UCLA, UNITED STATES

## Abstract

Previous studies suggest stable and robust control of a brain-computer interface (BCI) can be achieved using electrocorticography (ECoG). Translation of this technology from the laboratory to the real world requires additional methods that allow users operate their ECoG-based BCI autonomously. In such an environment, users must be able to perform all tasks currently performed by the experimenter, including manually switching the BCI system on/off. Although a simple task, it can be challenging for target users (e.g., individuals with tetraplegia) due to severe motor disability. In this study, we present an automated and practical strategy to switch a BCI system on or off based on the cognitive state of the user. Using a logistic regression, we built probabilistic models that utilized sub-dural ECoG signals from humans to estimate in pseudo real-time whether a person is awake or in a sleep-like state, and subsequently, whether to turn a BCI system on or off. Furthermore, we constrained these models to identify the optimal anatomical and spectral parameters for delineating states. Other methods exist to differentiate wake and sleep states using ECoG, but none account for practical requirements of BCI application, such as minimizing the size of an ECoG implant and predicting states in real time. Our results demonstrate that, across 4 individuals, wakeful and sleep-like states can be classified with over 80% accuracy (up to 92%) in pseudo real-time using high gamma (70–110 Hz) band limited power from only 5 electrodes (platinum discs with a diameter of 2.3 mm) located above the precentral and posterior superior temporal gyrus.

## Introduction

Brain-computer interface (BCI) systems enable communication between the central nervous system and an external device. This technology aims to restore cognitive and motor function in individuals with disability due to spinal cord injury, stroke, or a neuromuscular disorder. BCIs consists of 4 main components: a sensor to sample neural activity, a mapping of neural activity into commands, a device to execute the commands, and an operational protocol to govern how the user interacts with the device [1]. For BCIs to be clinically viable, all of these elements need to be carefully designed in concert so target users receive substantial improvement in function, reliable long-term performance (multiple years), and the ability to operate their device autonomously.

The translational requirements of BCI may be satisfied with systems that sample neural activity via electrocorticography (ECoG), a signal acquisition modality that detects changes in field potentials on the cortical surface [2,3]. In a recent study, Wang et al. used an online neural decoder to map ECoG signals from sensorimotor cortex to the velocity of a computer cursor in a closed-loop center-out task. The subject, a human with tetraplegia, was able to achieve robust real-time control of the cursor in 3 dimensions after just over a week of training [4]. Furthermore, there is evidence demonstrating that ECoG signal quality and the maps of ECoG signals to kinematic parameters are stable over long durations (months) [5]. Taken together, these findings suggest ECoG can stably and effectively be mapped into device commands such that target users receive long-term functional benefit.

The next step in the development of clinically viable ECoG-based BCIs is to create methods that enable autonomous operation of this technology in real world scenarios. In such an environment, the user must be able to autonomously perform all of the essential tasks usually completed by the experimenter, such as manually switching the BCI system on/off and determining when BCI control is permitted. Although straightforward, these tasks are difficult for target users because of severe motor disability. Thus, there is a need for automated methods that can determine solely from the brain activity of the user when a BCI should be switched on and controlled.

**Fig 1:**
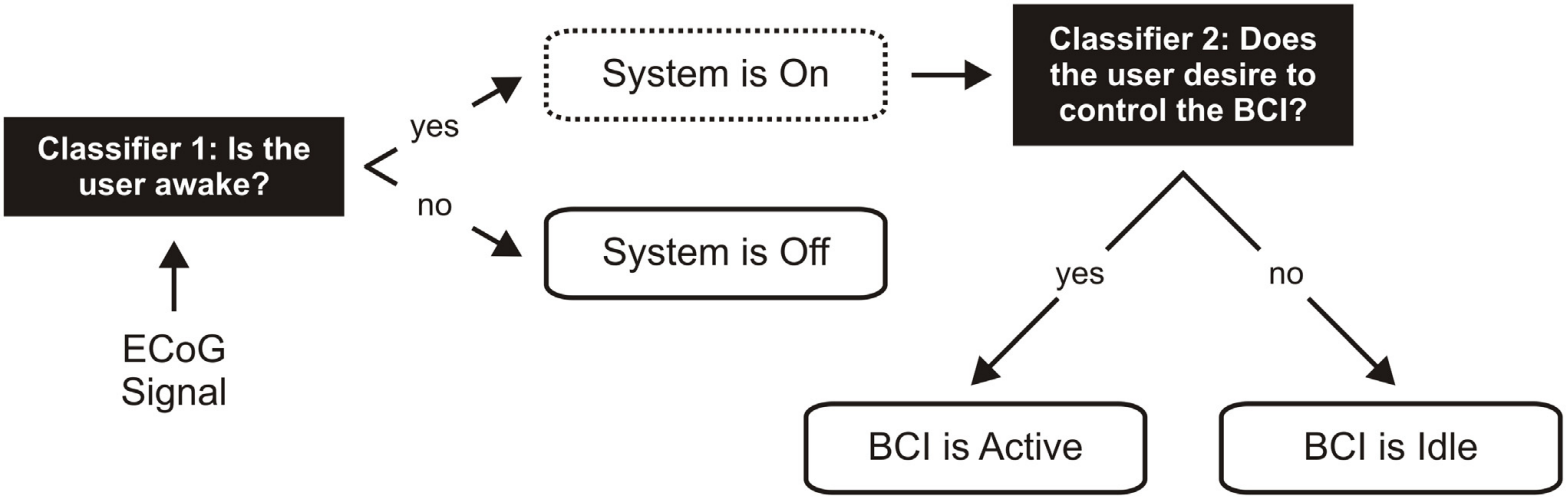
Autonomous operation of a BCI. Shown above is an illustration of how a System On/Off switch (classifier 1) and an asynchronous control mechanism (classifier 2) could be used to determine when an individual wants to control their neuroprosthetic. The BCI would be switched on and operational only when the individual is awake. While awake, an asynchronous control mechanism would be used to give complete control of the neuroprosthetic to the individual. The BCI would be switched off while the individual is asleep to prevent inadvertent activation of the neuroprosthetic.

The process of determining when a BCI should be controlled can be structured as a hierarchy (Fig 1). At the highest level of the hierarchy (classifier 1), the BCI system evaluates the level of consciousness of the user and decides whether the device should be turned on (fully conscious) or off (minimally conscious). Then, if in the ‘on’ state, the BCI system will move to the second layer in the hierarchy (classifier 2) and determine whether or not the user desires to control the device. Several studies have proposed using an asynchronous control mechanism for the second step in this process. Electroencephalographic (EEG) BCIs have used explicit motor imagery to switch between active/control and idle/no-control states [6,7]. BCIs that use single unit recordings have used automated detection of motor planning to separate these states [8]. Using ECoG in non-human primates, Williams et al. showed active/control and idle/no-control states could be reliably discriminated in real-time based on the amplitude of the alpha and beta signals from sensory cortex [9].

In this study, we propose a novel and automated method to decide when to switch the BCI on and off (classifier 1). We assumed a BCI will need to be switched on and ready for control when an individual is awake and switched off when an individual is asleep. Thus, our objective was to create a practical and automated method to reliably estimate when individuals are awake or asleep based on their ECoG activity. Many methods exist to delineate sleep and wake states using EEG, but they are not applicable in ECoG-based BCIs. Several studies have characterized the ECoG dynamics during sleep in humans [10,11,12,13,14,15,16], and methods have been created to differentiate states [16,17], but these studies did not account for the practical constraints of BCI application, such as minimizing the footprint of an invasive ECoG implant and to predict states in real-time. We built probabilistic models to estimate when an individual is asleep or awake given their ECoG activity. We constrained our models to determine the minimal number of ECoG electrodes needed to reliably estimate states. Our results suggest it is possible to accurately predict sleep-like and wakeful states in real-time using ECoG from a small region of cortex. We found the optimal cortical regions and spectral features to be consistent across subjects, which suggests our method may generalize to the target population and be a robust tool to turn a BCI on and off.

## Methods

### Ethics Statement

This study was approved by the Human Research Protection Office at Washington University School of Medicine. All patients gave informed written consent prior to participation.

### Participants

**Fig 2:**
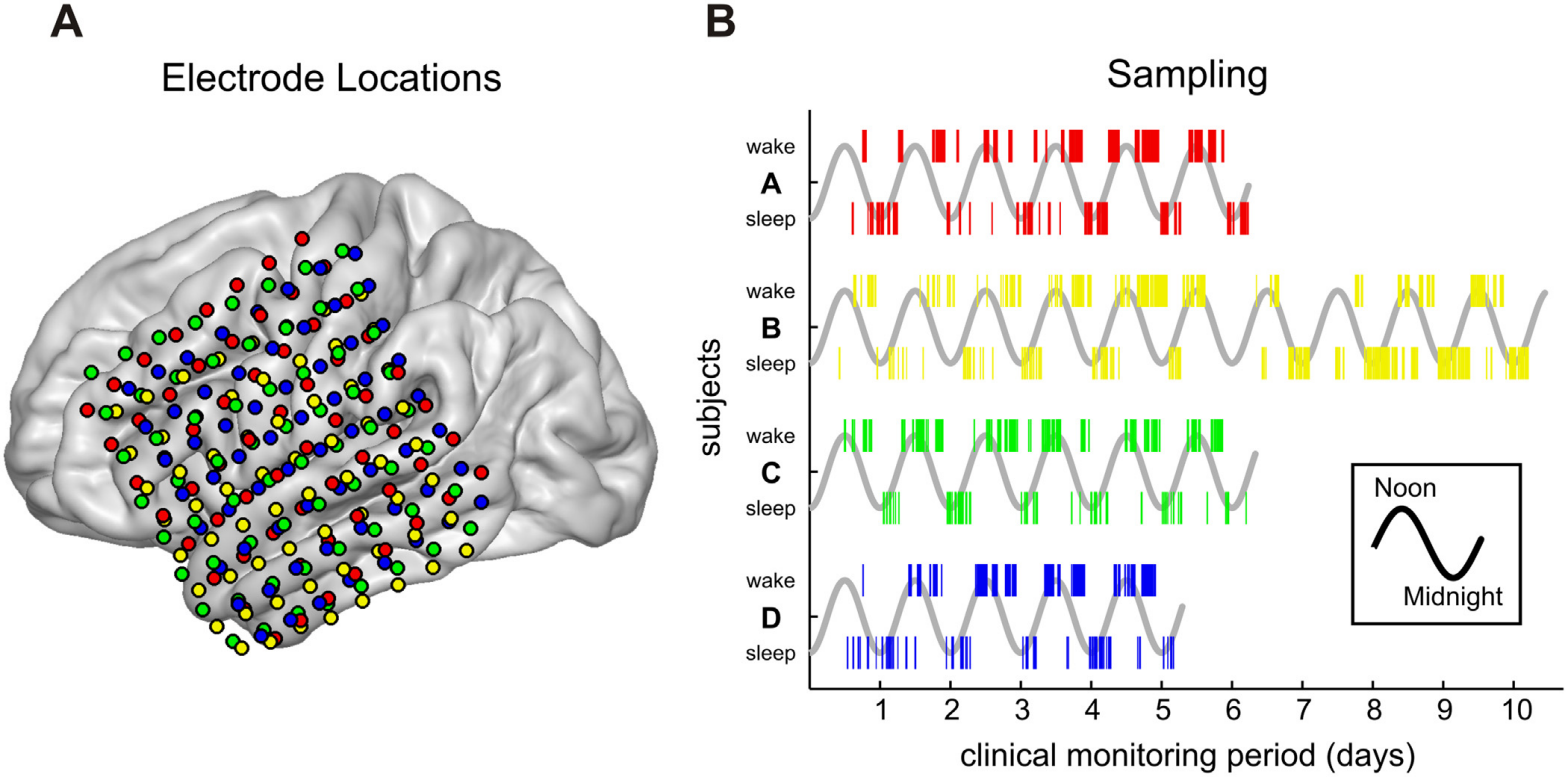
Electrophysiological and behavioral data collection. Electrocorticographic (ECoG) data were collected from four patients implanted with subdural 8x8 electrode grids over left frontal, temporal, and parietal areas of the cortex. The colors red, yellow, green, and blue signify subjects A, B, C, and D, respectively. (A) Electrode locations for each participant on an atlas brain. (B) Time and duration of behavioral epochs collected for analysis. The gray sine waves represent time oscillating between noon (peak) and midnight (trough) for the entire clinical monitoring period for each participant. The colored bars superimposed on top of the sinusoid indicate the time and duration during which a Wakeful (top row) or Sleep-like (bottom row) epoch was identified. Due to interruptions from seizure events, clinical care, and periods of uncertainty of cognitive state, behavioral epochs were sampled discretely.

Participants for this study were four human patients (two female) undergoing surgical treatment for intractable epilepsy at Barnes Jewish Hospital in Saint Louis, Missouri. Patients were temporarily implanted with subdural ECoG electrode grids to localize seizure onset foci. The location and number of electrodes were based solely on clinical need and are shown in panel A of Fig 2. The grids were constructed by PMT corporation and feature flat circular platinum electrodes with a 2.3 mm diameter and 10 mm inter-electrode distance. All electrodes were referenced to a skull-facing electrode of the same size. Patients were monitored for seizure activity for one to two weeks during which all studies were conducted. Relevant biographical information for each participant is displayed in Table 1.

**Table 1:** Patient demographics and clinical information.

Subject	Sex	Age	Handedness	Seizure Foci	Grid location	Wake data sampled (hrs)	Sleep data sampled (hrs)
A	F	44	Right	ATL	Left F/T/P	77.3	54.7
B	M	55	Right	LTL	Left F/T/P	66.7	78.6
C	F	21	Left	ATL	Left F/T/P	62.3	35.5
D	M	29	Right	PSTG	Left F/T/P	52.5	31.4

F, Frontal; T, Temporal; P, Parietal; LTL, Lateral temporal lobe; ATL, Anterior temporal lobe; PSTG, Posterior superior temporal gyrus.

### Behavioral Data

Participants were not directed to perform any particular task in this study. Rather, they were allowed to behave naturally and freely throughout the entire period of time they were implanted with electrodes. Video and audio recordings capturing subject behavior were analyzed to identify epochs when the subject clearly appeared to be either awake or asleep. ‘Wakeful’ epochs were identified as periods when the subjects had their eyes open and were cognitively active (talking, eating, listening, looking, and making voluntary movements, etc.). ‘Sleep-like’ epochs were identified as periods when subjects were quiescent, i.e., lying in bed with their eyes closed, breathing slowly, and making no volitional movements. Sleep-like epochs were not defined based on electrophysiological criteria nor further sorted into sub-states. The reason for using this method rather than using the gold standard detailed by Iber and colleagues[18] is twofold. First, since we are using Machine Learning methods to build state estimation models, we do not want feature selection in the models to be biased towards electrophysiological sleep staging criteria. It is critical that the state labels of the data are independent from the electrophysiological input to the state estimation models. Second, we are not concerned about estimating specific sleep stages since we are only in interested in studying states that are behaviorally relevant for an on/off BCI switch, which is whether an individual in general is awake or asleep. The capitalized terms ‘Wakeful’ and ‘Sleep-like’ will be used throughout the remainder of the paper to refer to the described states.

Recordings from all days except the final day of monitoring were used for constructing state estimation models. The final day of recording was used to quantify the efficacy of the state estimation models in real-time and across state transitions (see section 2.9). To avoid introducing bias from the observer uncertainty that occurred when subjects transitioned between states, only epochs where the behavioral state of each subject remained unchanged for at least 20 minutes (either completely Wakeful or Sleep-like) were used for analysis and training the models. This stringent epoch selection process helped ensure that our subjects were indeed in the identified state and allowed for ideal training of our state estimation models. Panel B of Fig 2 illustrates when during the monitoring period epochs were sampled and how long they lasted for each subject. Epochs that were behaviorally ambiguous were not used in the analysis.

### ECoG Recordings and Signal Processing

ECoG signals were recorded using Nicolet c128 amplifiers made by Natus Medical Incorporated and sampled at 256 Hz. Raw signals were high-pass filtered at 0.05 Hz using a 3^rd^ order Butterworth filter. The signals from all ECoG electrodes were visually examined for quality. Electrodes containing an excessive amount of noise or that were located above the clinically determined seizure foci were removed from further analysis. Additionally, time epochs containing epileptic activity or an artifact in a majority of electrodes were discarded. The mean of the non-noisy electrodes was regressed out of the signal from each electrode.

The power spectral density (PSD) of the ECoG signal from each electrode was estimated using Welch’s method [19]. The Welch’s windows had a width of 2 seconds (frequency resolution of 0.5 Hz) and a 50% overlap. Power spectra were consolidated into canonical frequency bands (delta: 0.1–4 Hz, theta: 4.5–8 Hz, alpha: 8.5–12 Hz, sigma: 12.5–15 Hz, beta: 15.5–25 Hz, low gamma: 25.5–50 Hz, and high gamma: 70–110 Hz) and then normalized by the total power across all frequency bands.

Differences in cortical electrophysiology between the Wakeful and the Sleep-like state were examined for each subject in the frequency domain using the sensitivity (or discriminability) index from signal detection theory [20]:

$$d_{b,c}^{\prime }=\frac{\mu_{b,c,Wake}-\mu_{b,c,Sleep}}{\sqrt{\rho_{Wake}\sigma_{b,c,Wake}^{2}+\rho_{Sleep}\sigma_{b,c,Sleep}^{2}}}\;,$$ (1)

where *μ*_*b*,*c*_ and *σ*_*b*,*c*_ are the mean band limited power (BLP) and the standard deviation of the BLP, respectively, across all epochs for the specified cognitive state at frequency band *b* and electrode *c*. *p* is the proportion of data belonging to each class.

### Logistic Regression Model for State Estimation

A logistic regression was employed to build models that could accurately predict the Sleep-like and Wakeful states given the ECoG signals. The ECoG signals from each behavioral epoch were broken into 120 second non-overlapping segments or instances. The PSD was calculated for each instance and consolidated into frequency bands using the method described in section 2.4, resulting in a set of features, ***p*** ∈ ℝ^*CxB*^, where *C* is the number of electrodes and *B* is the number of frequency bands. The features and the class labels, *y* ^(*i*)^ (-1 for Sleep-like or +1 for Wakeful), for all instances from a particular epoch were randomly placed as a group into either a training or a test set such that class distribution was preserved in each set and so approximately 80% of the total number of instances across all epochs were in the training set (approximately 20% in the test set). Five-fold cross validation was used to learn the models. Each fold had a unique test set.

Within a fold, each feature was centered by the feature mean across all training instances and normalized by the Euclidean norm of the feature across all training instances:

$$x_{b,c}^{\left(i\right)}=\frac{p_{b,c}^{\left(i\right)}-\;{\bar{p}}_{b,c}}{\sqrt{\sum_{i=1}^{n}{\left(p_{b,c}^{\left(i\right)}-\;{\bar{p}}_{b,c}\right)}^{2}}}\;,$$ (2)

where $p_{b,c}^{\left(i\right)}$ is the BLP of instance *i* at frequency band *b* and electrode *c*, and ${\bar{p}}_{b,c}$ is the average BLP over the training set within a fold. The feature mean and norm calculated from the training set were also used to center and normalize the test set within the fold.

Models were learned using all features, ***x*** ∈ ℝ^*CxB*^, and also using a subset of features, ***x***_b_ ∈ ℝ^*Cx1*^, i.e., a unique model was learned for the group of features belonging to each frequency band. For each training set, $R_{b}=\left\{x_{b}^{\left(1\right)},\ldots ,x_{b}^{\left(n\right)};y^{\left(1\right)},\ldots ,y^{\left(n\right)}\right\}$ or *R* = {***x*** ^(1)^,…,***x*** ^(n)^;*y* ^(1)^,…,*y* ^(n)^} of *n* instances for a single patient, we modeled the probability that a patient was in the Wakeful or Sleep-like state for instance *i* using a linear model transformed by the sigmoid function, commonly referred to as logistic regression:

$$\text{Pr(}y^{\left(i\right)}\;|z^{\left(i\right)};w)=\frac{1}{1+e^{-y^{\left(i\right)}w^{T}z^{\left(i\right)}}}\;,$$ (3)

where ***z*** is either ***x*** or ***x***_*b*_, and ***w*** is a weight vector that parameterizes our model. We solved for these weights by maximizing the probability that each reading was predicted correctly:

$$\underset{w}{\text{max}}\;\;\prod_{i=1}^{n}\text{Pr(}y^{\left(i\right)}\;|z^{\left(i\right)};w)$$ (4)

Or, equivalently, we can solve for ***w*** by minimizing the sum across instances of the negative logarithm of the probability:

$${\text{min}}_{w}\Sigma_{i=1}^{n}log\left(1+e^{-y^{\left(i\right)}w^{T}z^{\left(i\right)}}\right)$$ (5)

Modeling the probability allowed prediction uncertainty to be represented naturally, which has practical value for BCI applications because it is safer if the BCI remains off when the system is uncertain of the user’s cognitive state.

Optimal cortical locations for estimating the Wakeful and Sleep-like states were identified by constraining the optimization problem. By adding an *ℓ*_*1*_ */ℓ*_2_ mixed-norm of the feature weights, we forced the learning algorithm to converge on a solution that uses BLP from all frequency bands, but from a sparse set of electrodes. The *ℓ*_*1*_ */ℓ*_2_ mixed-norm regularized logistic regression is shown below:

$${\text{min}}_{w}\sum_{i=1}^{n}\text{log}\left(1+e^{-y^{\left(i\right)}w^{T}x^{\left(i\right)}}\right)+\lambda \;\sum_{c=1}^{c}\sqrt{\sum_{b=1}^{B}{\left(w_{b,c}\right)}^{2}}\;,$$ (6)

where *λ ≥ 0* trades off prediction accuracy on the training set with electrode weight sparsity. Similarly, for the models only utilizing features from one specific frequency band as an input, ***x***_*b*_, we employed an *ℓ*_1_-regularized logistic regression model:

$${\text{min}}_{w}\Sigma_{i=1}^{n}\text{log}\left(1+e^{-y^{\left(i\right)}w^{T}x_{b}^{\left(i\right)}}\right)+\lambda ∥w{∥}_{1}$$ (7)

Electrode sparsity was independently varied from 1 to 20 electrodes. The corresponding hyper-parameter *λ* was learned using a binary search on the training set of each fold. Initially, an arbitrary value was assigned to *λ*, and a subsequent model was constructed. If the model was more sparse than desired, then *λ* was decreased to reduce the impact of the constraint on the model. Conversely, if the model was less sparse than desired, then *λ* was increased. This process was systematically repeated until *λ* converged on a value that provided the desired electrode sparsity in the model.

The output of each model was the probability that the subject was in the Wakeful state (Eq 3; *y* ^*(i)*^ = 1). Thus, the state was estimated using the following rule:

$$State\;Estimation\left(i\right)=\;\{\begin{array}{c} Wakeful;\text{Pr(}y^{\left(i\right)}=1|x^{\left(i\right)};w)\geq 0.5\\ Sleep\text{-}like;\text{Pr(}y^{\left(i\right)}=1|x^{\left(i\right)};w)<0.5 \end{array}$$ (8)

Model performance was quantified by evaluating the accuracy, sensitivity, and specificity on the test set of each fold.

The final day of ECoG recordings for each subject were not used to construct models. Instead, these data were used to evaluate each model’s ability to estimate states and state transitions in pseudo real-time (every 10 seconds). The ECoG signals from the entire day were broken down into 120 second segments using a sliding window that shifted 10 seconds at a time. Normalized BLP was calculated for each of these segments using the methods described in sections 2.4 and 2.6. Each instance was assigned a class label according to the methods in section 2.3. Instances within state transitions where the behavioral state was visually ambiguous were assigned the label of the previous instance. The feature weights used to estimate the state of each segment were calculated for each model by retraining the models on the entire offline data set.

### Construction of Subject-averaged Cortical Maps

Cortical maps were made using a strategy adapted from the methods presented by Hermes et al. in 2010 [21]. Each subject’s cortex was reconstructed as a surface using a pre-operative T1-weighted MRI, and the location of each subject’s electrodes was determined using a post-operative CT scan. The electrodes and the reconstructed cortical surface were co-registered to the same atlas space for all subjects. The co-registration had an intrinsic error because the electrode locations were found relative to the compressed post-operative brain, whereas, the reconstructed brain surface was made using images of an unperturbed pre-operative brain. This error was corrected by projecting the electrodes onto the pial surface of the reconstructed brain following a vector normal to the local electrode grid plane. This projection was done while enforcing the constraint that the inter-electrode distances remained at 10 mm.

Metrics were mapped onto each subject’s brain surface using Gaussian spheres that were centered at the location of each electrode in space and scaled by the particular metric that was being mapped. Each face on the cortical surface mesh was assigned a value according to where it intersected the field created by summing all of the Gaussian spheres in space. The cortical surface mesh for each subject had the same number of faces and was designed so that a particular face corresponded to homologous anatomy across subjects. A subject-average map was made by taking the mean at each face across subjects. The subject-average maps were displayed on an atlas brain, which was constructed by averaging the structural MRI of brains of 38 healthy individuals. The density of sampling on the cortical surface was regressed out to eliminate regional sampling bias.

## Results

### Spatio-spectral Differences between States

**Fig 3:**
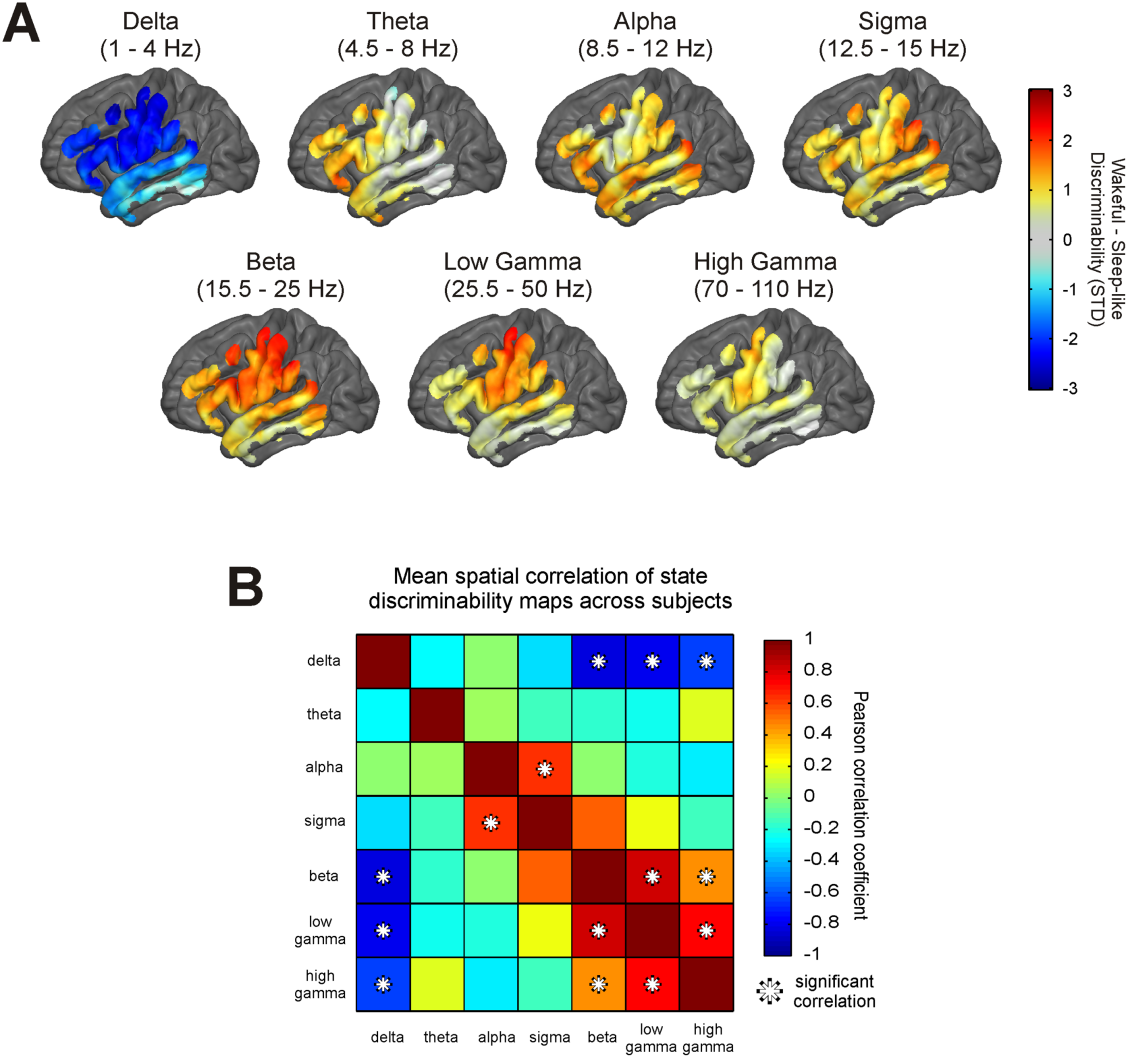
Discriminable spatio-spectral features between the Wakeful and Sleep-like states. Panel A shows the topographic heat maps of the average discriminability in band limited power (BLP) between the Wakeful and the Sleep-like states. Warm colors indicate cortical areas where the mean of the BLP distribution on average was greater in the Wakeful state, whereas, cool colors indicate cortical areas where the mean of the BLP distribution on average was greater in the Sleep-like state. Silver areas specify locations where BLP distributions were not discriminable between states, and dark charcoal areas signify locations where an ECoG signal was not sampled from. Panel B illustrates significant relationships (p < 0.00238; Bonferroni corrected) between the topography of the BLP discriminability maps. The relationship between two maps was quantified using a Pearson spatial correlation. Significant positive correlations were found between maps for alpha and sigma, beta and low gamma, beta and high gamma, and low gamma and high gamma. Significant negative correlations were observed between maps for delta and beta, delta and low gamma, and delta and high gamma.

The discriminability (Eq 1) in BLP between the Wakeful and Sleep-like states at each electrode was mapped onto each subjects’ reconstructed cortical surface. Fig 3A displays the subject average map for each frequency band. Only cortical locations sampled by at least 2 subjects are shown. Warm colors indicate regions where the mean BLP is greater in the Wakeful state than in the Sleep-like state on average, and cool colors indicate the opposite. The intensity of the color conveys in units of standard deviations how separated the distribution of BLP is between states. As seen in the figure, the mean of the delta power distribution is greater in the Sleep-like state than in the Wakeful state at almost all sampled cortical locations on average with maximal discriminability occurring at frontal and parietal locations. All other frequency bands appear to have either a greater mean power in the Wake state or no discriminability in power distributions between states.

The topographies varied across frequency bands. Fig 3B illustrates statistically significant relationships between the different maps. Spatial correlations were calculated between each BLP discriminability map within subject. A one-way t-test was performed to identify significant relationships across subjects. Significant relationships (p < 0.00238; Bonferroni corrected for multiple comparisons) include negative correlations in topography between delta and beta, delta and low gamma, and delta and high gamma. Significant positive correlations in topography were found between alpha and sigma, beta and low gamma, beta and high gamma, and low gamma and high gamma. This result suggests that the cortical locations where delta power can discriminate states are the same cortical locations where the higher frequencies (> 15 Hz) can discriminate states. The next step is to determine which of these locations and which frequencies are the most reliable at differentiating the Wakeful and Sleep-like states.

### Optimal Spatio-spectral Features for State Estimation

**Fig 4:**
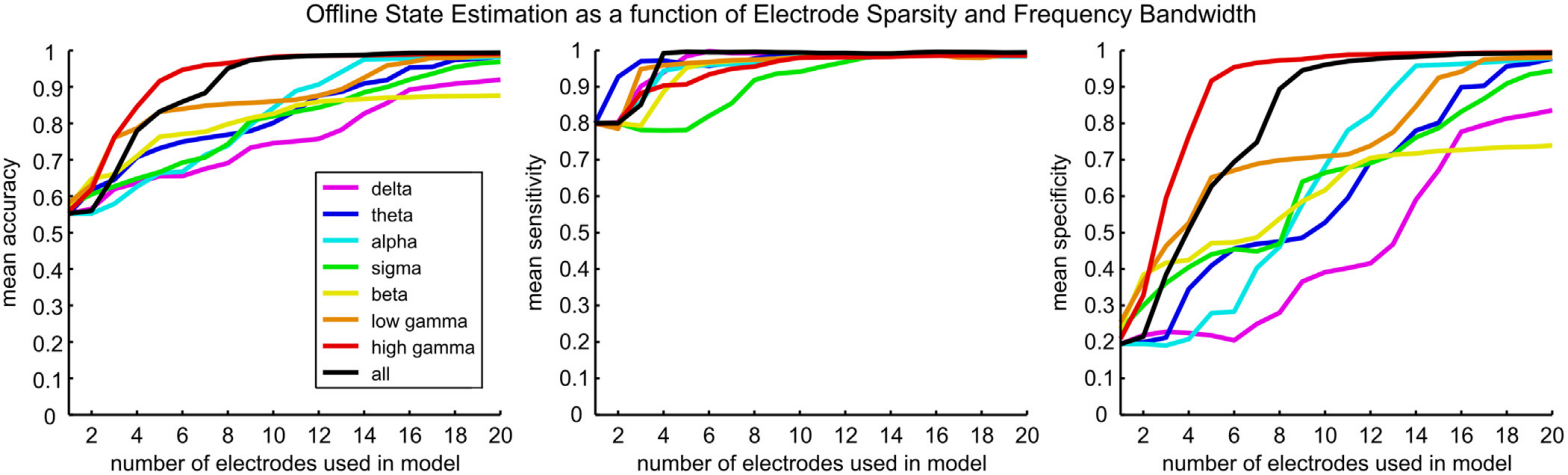
Model performance as a function of electrode sparsity. The mean accuracy, sensitivity, and specificity are shown for the subject average. Each color denotes the performance of a different frequency band, which is illustrated in the legend in the bottom right of the first plot. Each metric increased monotonically for each frequency band as signals from more electrodes were included in the model. The high gamma band (red) and all frequency bands (black) were the most informative across subjects, as on average they required signals from the least number of electrodes in order to reliably estimate cognitive state.

For each subject, models were trained to estimate states based on the BLP of the ECoG signal. The number of electrodes and the spectral range of the features used in each model were independently varied. The accuracy, sensitivity, and specificity of each model were evaluated on a test set of fixed-state samples. Fig 4 displays the average of these quantities across subjects. All models demonstrated high sensitivity (detection of the Wakeful state). Performance was distinguished between models based on specificity (detection of the Sleep-like state). Having high specificity is essential for a BCI on/off switch to avoid episodes when a BCI is inadvertently activated. Additionally, it is critical to estimate states using signal from the least number of electrodes because it is desirable to minimize the size of an ECoG implant. The models using only high gamma BLP features required ECoG signal from the fewest number of electrodes to achieve a high specificity. The overall balance between sensitivity and specificity is reflected in the mean accuracy. Models using high gamma BLP were the most accurate, and on average needed signal from only 5 electrodes to correctly classify over 90% of instances.

Interestingly, the model using signals from all frequency bands on average did not estimate state as well as the high gamma band model, even though the same high gamma features were present in the feature space (see Fig 4). Contrary to our expectation, the combination of information from different frequency bands did not improve state estimation. Most likely this result is an effect of the different regularization terms implemented in the model regression. The *ℓ*_*1*_ */ℓ*_2_-mixed norm utilized in the all frequency bands model grouped together BLP features for all frequencies from a particular electrode. An *ℓ*_1_-norm was applied across groups to identify features within a sparse set of electrodes that best estimated states. Within a group, or electrode, an *ℓ*_2_-norm was applied, which encouraged feature weights across frequency bands to be small and relatively equal in magnitude. This preference for small equal weights resulted in the model valuing information from each frequency band evenly, which may be why information in the high gamma signal did not overcome any noise present in the other frequency band features.

**Fig 5:**
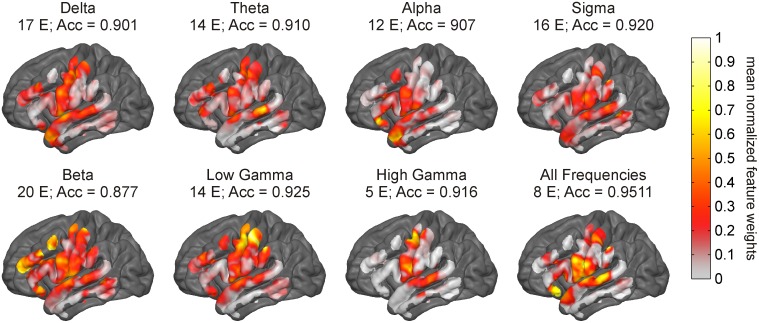
Important cortical areas for cognitive state estimation. Shown above is the mean projection of the normalized electrode weights from the lowest order model able to estimate state with an accuracy (Acc) ≥ 0.9 for each type of feature space. The models built using only the high gamma band BLP performed the best, with signals acquired on average from 5 electrodes above the precentral and posterior superior temporal gyrus. Other frequencies showed some regional specificity across subjects but overall required sampling of broad regions of the cortex to make reliable predictions.

The weights of the electrodes used in each model were projected on each subject’s cortical surface reconstruction to identify the cortical locations that were most important for estimating state. Each subject’s maps were normalized by their maximum weight and then averaged across subjects to distinguish the optimal cortical regions. Fig 5 displays the subject average map for each frequency band for the model that used the least number of electrodes and had an average accuracy ≥ 0.9. The high gamma BLP, which was the most effective signal for estimating states, was sampled on average by only 5 electrodes located above the precentral gyrus and posterior superior temporal gyrus. The model using signals from all frequency bands performed similarly, utilizing signals from 8 electrodes clustered primarily above inferior sensorimotor areas, posterior superior temporal gyrus, and the inferior frontal gyrus. The remaining models, built using features from the other frequency bands (delta to low gamma), sampled BLP from at least twice as many electrodes as the optimal high gamma band model to achieve a similar quality of state estimation. Electrode feature weights for these models displayed some clustering over specific areas of cortex, but overall were diffusely spread across the sampled cortical surface.

### Pseudo Real-time State Estimation

**Fig 6:**
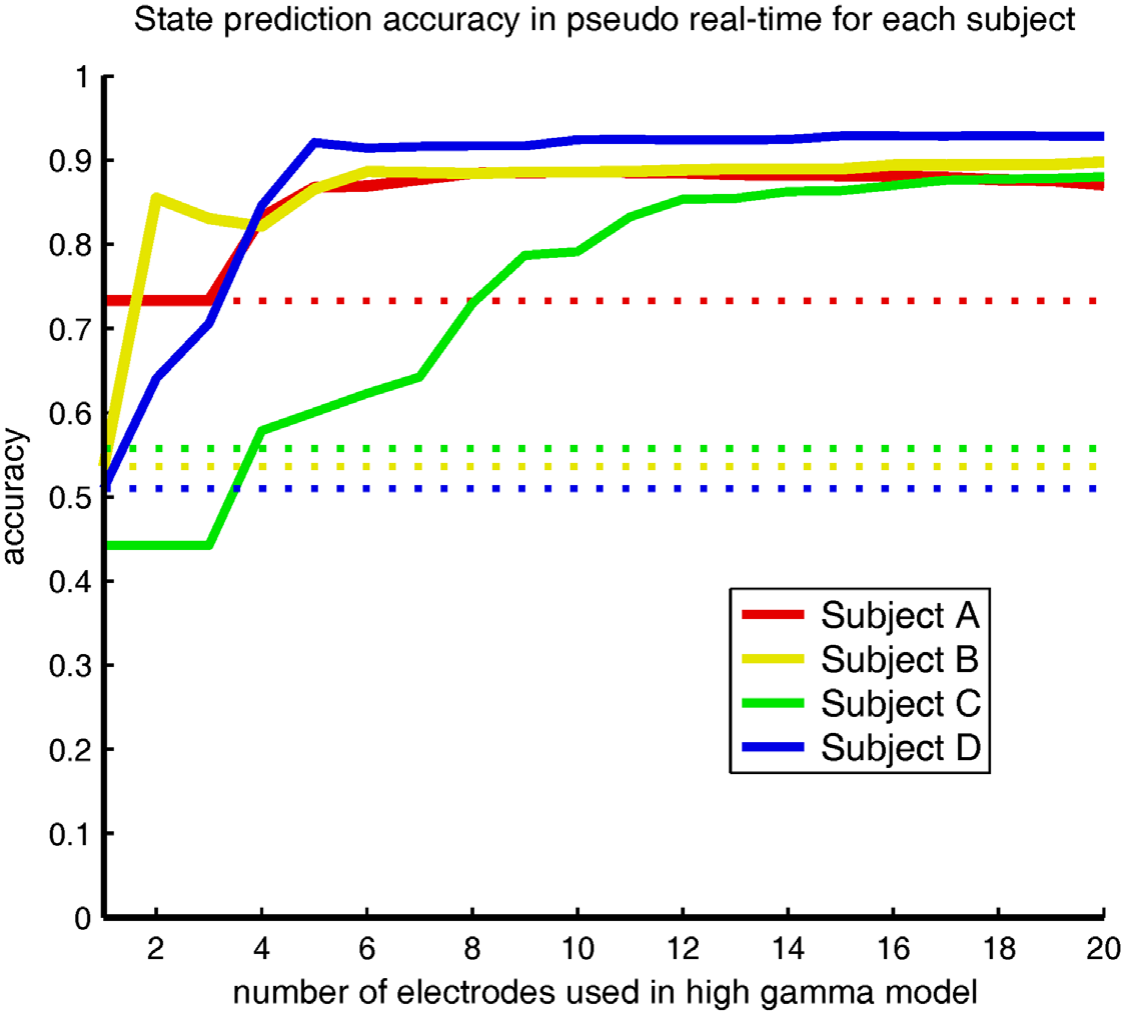
Prediction accuracy of high gamma band models in pseudo real-time on a novel signal that naturally transitions between states. Solid lines indicate accuracy as a function of the number of electrodes and dotted lines indicate chance levels. Accuracy of online state estimation for subjects A, B, and D is comparable to the prediction accuracy of the high gamma band models in the offline analysis.

Fig 6 illustrates the accuracy of the high gamma band models for each subject when evaluated in pseudo real-time (prediction every 10 seconds) on a novel signal that naturally transitions between states. As seen in the figure, the online accuracy of the high gamma band models for three subjects (A, B, and D) is on par with the offline training accuracy (Fig 4). Using high gamma band signal from only 5 electrodes, states are predicted online with an accuracy of 86.7%, 86.5%, and 92.1% for subjects A, B, and D, respectively. On the other hand, the high gamma band models for subject C performed poorly (60.1% accuracy using 5 electrodes), although still above chance (dotted line in Fig 6) when using signal from 4 or more electrodes. Interestingly, the high gamma band models for subject C had a very high offline training accuracy, which suggests that models may have been over-fit during training.

**Fig 7:**
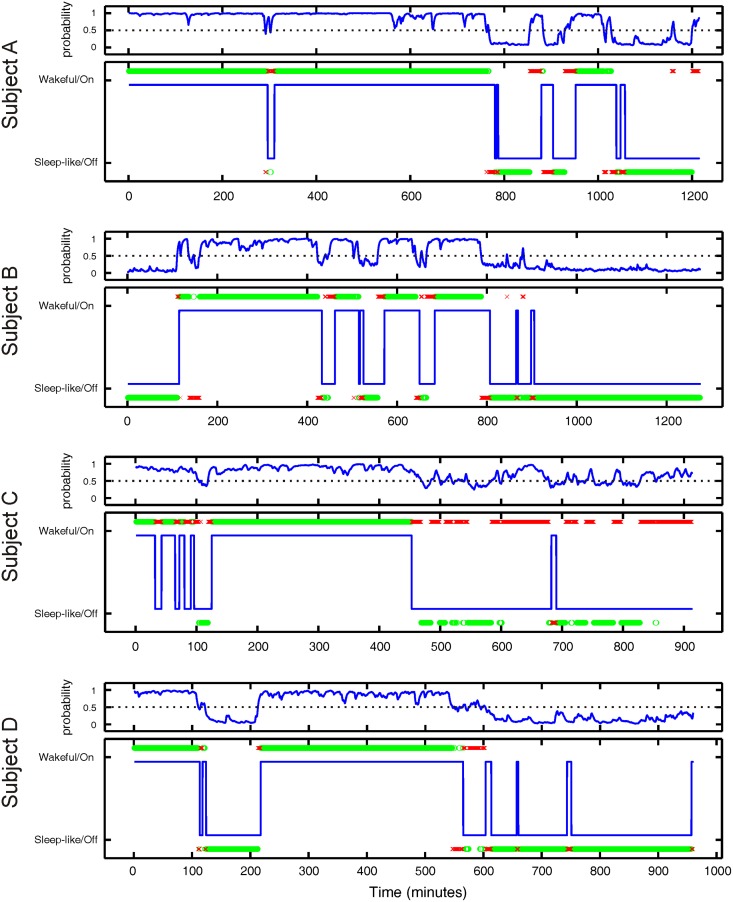
Pseudo real-time state estimation of state transitions. Depicted in the top panel for each subject is the output of the 5 electrode high gamma band model in pseudo real-time on a never-before-seen test set of data that includes natural transitions between the Sleep-like and Wakeful states. The output is the probability each subject is in the Wakeful state given the weighted high gamma BLP signal from 5 electrodes. The dashed line is the decision threshold of the model. The lower panel for each subject illustrates the visually identified behavioral state (blue line) and the state predictions made by the model. The green circles are instances where the model prediction matches the visual classification and the red x’s are instances where the model prediction does not match the visual classification. A prediction is made every 10 seconds using the previous 120 seconds of signal. Most incorrect predictions occurred near state transitions.

The detailed time-course of the predictions from the 5 electrode high gamma band model are illustrated for each subject in Fig 7. This result provides insight on why states were estimated poorly for subject C and where prediction errors were made for other subjects. The model output, or the probability of the subject being in the Wakeful state, is shown in the top panel for each subject. A threshold of 0.5, where the probability of the subject being in the Wakeful or Sleep-like state is equal, was applied to the output to determine state prediction, which is shown in the bottom panel for each subject. As seen in the figure, the incorrect state predictions (red x’s) for subjects A, B, and D primarily occurred near state transitions. This was expected since the behavioral classification of state transitions using only visual evidence was an imprecise process and susceptible to observer bias (see methods section 2.3 and 2.9). Looking at the predictions for subject C, we observe that the majority of misclassifications occurred in the second half of the time frame when the subject appeared to be asleep.

## Discussion

It is well understood that cognitive states (Wakefulness, NREMS, and REMS) have distinct spectral topographies [22,23,24,25]. Our results corroborated these findings (Fig 3) and demonstrated that, given sufficient cortical sampling, BLP from all canonical frequency bands can accurately estimate the Wakeful and Sleep-like states (Fig 4). However, by seeking out a sparse set of features within the ECoG signal, we found that the high gamma BLP was the most informative signal. On average, over 90% prediction accuracy was achieved when estimating discrete state epochs offline using high gamma BLP sampled from 5 electrodes located above precentral and posterior superior temporal gyrus on the left hemisphere. In pseudo real-time, over 80% accuracy (as high as 92.1% in subject D) was achieved using the same high gamma band models.

Previous studies in humans have shown that the mean power in the higher frequencies (> 35 Hz), regardless of cortical location, is greater in a wake state compared to the REM and NREM sleep states, although the relative magnitude of the gamma power varies depending on cortical location [22,26]. This potentially explains why the high gamma signal was able to reliably differentiate the Sleep-like and Wakeful states in this study. A more specific strategy for estimating states is apparent when considering the optimal electrode locations. Work by Crone et al. has shown that an increase in focal high gamma BLP in inferior and superior precentral gyrus is correlated to the onset of sustained activity in the tongue and contralateral arm, respectively [27]. Similarly, high gamma BLP increases in left superior temporal gyrus are correlated to auditory and language processing [28]. Taken together, the result suggests our mechanism for delineating the Wakeful and the Sleep-like state is to detect for the presence of motor, auditory, and language activity. This finding is intuitive, as motor, speech, and auditory processing occur more frequently and are more robust while an individual is awake rather than asleep. This strategy is potentially problematic for BCI application since most target users (individuals with severe motor disability) are not able to generate volitional movements. We are unable to say if the high gamma BLP features would be optimal in individuals with motor disability, but there is substantial evidence demonstrating that motor-related cortical activations can be generated in this demographic as a result of imagined movements [29,30], and subsequently utilized for BCI control [4]. Additionally, the cortical distribution of ECoG activity is similar between imagined and actual movements [31]. Thus, we conjecture that cortical activations associated with imagined motor activity may be used to differentiate the Wakeful and Sleep-like states in individuals with motor disability, although future studies will need to be conducted to confirm this hypothesis.

Given the suggested mechanism, it is plausible that other spectral features are equally capable as the high gamma band at differentiating cognitive states. The same studies by Crone et al. that demonstrated high gamma power augmentation during motor, auditory, and language processing also showed correlated changes in alpha, beta (only in motor tasks), and low gamma power. These features were generally less robust, more diffuse across the cortical surface, and were modulated at a slower time scale [27,28,32]. The lack of specificity, both spatially and temporally, may be what resulted in substandard state estimation for those features in our study. Additionally, there are features we were unable to sample that presumably would be useful for cognitive state estimation, e.g., high gamma band activity in visual areas in the occipital lobe. Tallon-Baudry et al. showed that gamma activity increases in occipital areas during both bottom-up and top-down processing of a visual search task [33]. This behavior occurs more frequently while awake and thus could potentially be useful for discriminating the Wakeful and Sleep-like states. There likely are other such features, but we are unable to assess them, given the limited electrode coverage over the left frontal, temporal and parietal regions.

For 3 out of 4 subjects, the high gamma band models were remarkably able to predict states in pseudo real-time with similar accuracy as in the offline analysis, even though there were more potential sources for error in the online case. In large part, these errors may be attributed to uncertainty in the visual classification (ground truth) of the state transitions. It was difficult to discern from the visual evidence precisely when the subjects had fallen asleep or awakened, as this process generally occurred over several minutes. Additionally, the period of wakefulness immediately following sleep where individuals have impaired cognitive performance and decreased arousal, known as Sleep Inertia, is characterized by EEG correlates that differ from the typical wakeful EEG [34,35]. Thus, for periods like the brief Wakeful epochs seen between 600 and 800 minutes for subject D in Fig 7 (subject D was woken in the middle of the night by nurses for clinical care—such disruptions were a frequent occurrence for all subjects), the model incorrectly predicted that the subject was in the Sleep-like state. This type of misclassification counted as a false negative in our analysis, but it has practical value, as it is safer and desirable for the users of a BCI to have their neuroprosthetic switched off if they are cognitively impaired even though they are awake.

The cognitive state estimation models were built and evaluated on human subjects with epilepsy, and it is well known that epilepsy disrupts normal sleep patterns and that antiepileptic drugs may also have an effect [36]. Moreover, the sleep-wake cycle is known to influence epileptic activity [36], which can manifest as gamma activity near the seizure focus[37]. Thus, there is a possibility that changes in epileptic activity were correlated to changes in cognitive state, which may have affected the feature selection for the state estimation models. Given that all electrodes deemed by clinicians to be above the seizure foci or containing epileptic artifacts were removed prior to our analysis, this potential confounder is unlikely to have affected the results.

A limitation of this study is that no BCI task was performed by the subjects. The results provide an initial demonstration that a limited area of cortex can be used to detect the Sleep-like and Wakeful states. A future study will need to be conducted to determine if our proposed mechanism to switch a BCI on/off would function in concert with an asynchronous BCI task. Nevertheless, our method shows promise for BCI applications, as the optimal sensorimotor [2,31,38,39] and language-related [40] cortical locations are also utilized in a number of ECoG BCI studies to control a neuroprosthetic device. The complementary results suggest that a BCI can both be controlled and switched on/off using neural activity sampled by a small ECoG implant placed in the left hemisphere over the sensorimotor region and posterior superior temporal gyrus.

## Conclusions

In this study, we demonstrated in 3 out of 4 subjects that high gamma BLP, sampled from as few as 5 subdural ECoG electrodes located above the precentral and left posterior superior temporal gyrus, can accurately estimate if a person is awake or asleep. This finding suggests that an efficient strategy for delineating a Wakeful state from a Sleep-like state is to detect for motor, auditory, and speech processing. Additionally, this result has practical application in ECoG-based BCI systems as a switch to turn a BCI on and off. This finding complements many existing BCI studies that use similar cortical regions for neuroprosthetic control. This result is a step towards providing autonomy to users and making ECoG-based BCIs a clinically viable technology.
